# Supplementary material for: Urban Green Spaces and Vector-Borne Disease Risk in Africa: The Case of an Unclean Forested Park in Libreville (Gabon, Central Africa)
Source: Int J Environ Res Public Health. 2023 May 10;20(10):5774. doi: 10.3390/ijerph20105774 (PMC10217798; doi:10.3390/ijerph20105774)
Supplement: Supplementary file 1 [file ijerph-20-05774-s001.zip › ijerph-2252703-supplementary.pdf]

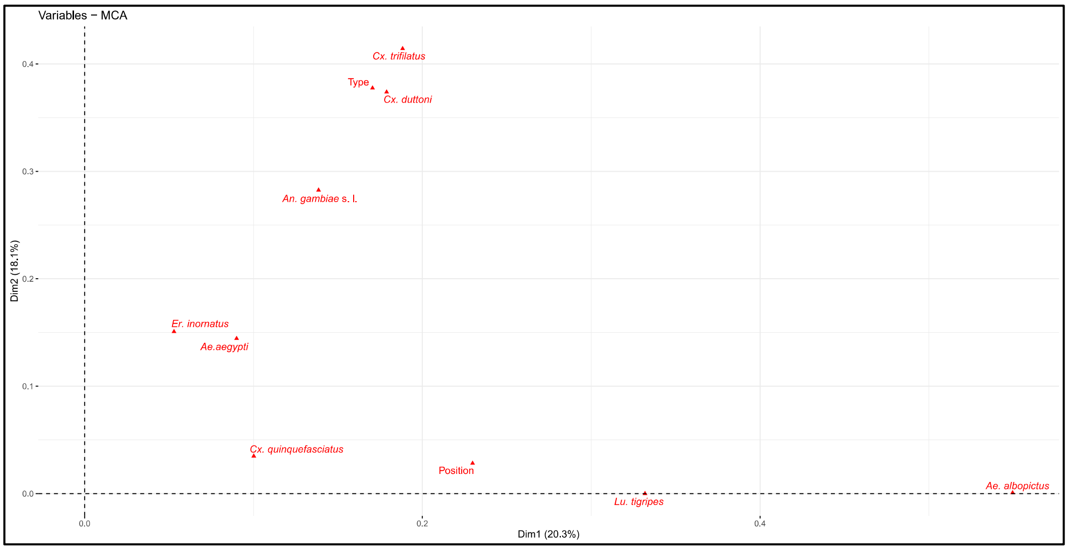

Figure S1: The bi-dimensional representation of environmental and biotic variables associated with larval habitat distribution on the factorial plan of the two first MCA dimensions (38.4% of explained inertia). The wide segregation of variable on the factorial plan translates a good correlation of these variables with the species composition variability among larval habitats.

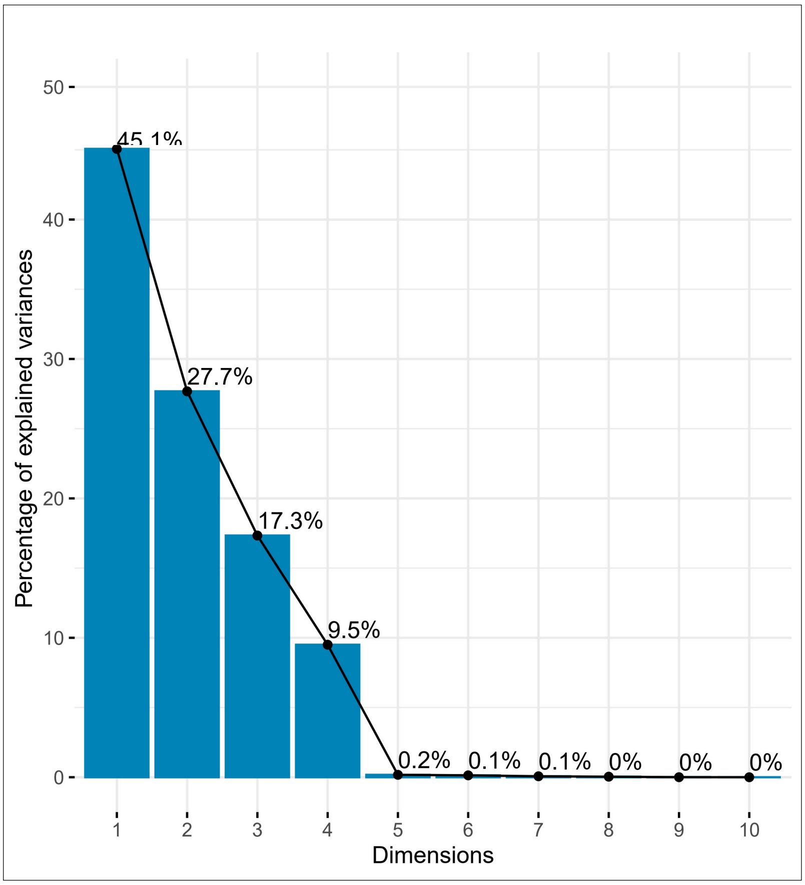

Figure S2: PAC's percentage of explained variances of data following the dimensions. The first two dimensions (1 & 2) refer to an explained variance of 72.8%.
